# Fifty Thousand Pounds

**Published:** 1891-04-04

**Authors:** 


### The Doctor's Armchair.

### FIFTY THOUSAND POUNDS.

King Solomon had a profound appreciation of the value of money, and King Solomon was one of the most far-seeing men that the very far-seeing nation of the Jews has ever produced. According to Solomon, there are two things of prime importance in this world, and money is one of them. "Wisdom is a defence," said Solomon, "and money is a defence." It is plain that Solomon meant that the man who is possessed of either wisdom or money is exceedingly well prepared for the whole campaign of life. Common experience entirely coincides with Solomon's. The man of money can hold his own with very little brains; and the man of brains can hold his own with very little money. It does not require ten minutes' reflection to convince a man who is accustomed to think that wisdom and money do, in an important sense, divide the world between them.

Here let us rein up our Pegasus, and offer a few explanatory words. A rash person might suppose, from what has been said, that the writer has an inordinate appreciation of money. Not at all! On the contrary, he believes that wisdom is the most precious treasure which this world has to offer; and that of all the varieties of wisdom, divine wisdom is the worthiest. But, if a man chooses to make a journey to the North Pole, it does not, therefore, follow that he must despise the South; and if a lady shows an extreme partiality for pretty bonnets, it is not necessary to conclude that she will have a preference for ugly boots. There is no particular necessity to cultivate extremes; and if a man have a high regard for wisdom, there is no reason whatever why he should have a high contempt for money. On the contrary, the really wise man will value money, whether he himself possesses it or not—because, whatever may be said or thought to the contrary, money is a powerful resource, and, in itself, perfectly harmless; and if there be brains and character to make use of the resource, it is astonishing what good results can be produced.

"All this preamble about fifty thousand pounds!" scoffs the millionaire. Yes! but just as a few British officers commanding a thousand Egyptian troops can defeat many Arabs commanding ten thousand dervishes, so can two or three men of first-rate business capacity produce larger and better results with fifty thousand pounds than the wooden-headed millionaire produces with twenty times fifty thousand. Here, for example, is one thing which fifty thousand pounds will do; it will bring in two thousand a-year; and two thousand a-year will by itself pension a hundred nurses for life with twenty pounds a-year each. But if it is used as a reinforcement instead of as a substantive army it will add five pounds a-year each to the pensions of four hundred nurses.

But this is one of the very smallest of the many things which fifty thousand pounds can do. Suppose we look at it as a seed corn! A good many nurses have been brought up in the country and know very well the life history of a seed corn. Take a grain of wheat for example, and put it into the soil on an October day. At Christmas it will be a green blade beneath the snow; in May it will be a strong stem rising a foot above the ground; in August it will be a golden ear bending downwards with the weight of its own rich fruit. We put one grain into the earth in October. Let us count the separate grains in this yellow ear: not one, nor twenty, nor fifty, but a hundred beautiful grains lie, tumbling over one another in our palm. Does anybody suppose that money cannot be made to fructify as well as wheat? It can, indeed, if the proper means be used. The writer knows far more than one man who has turned his tens into hundreds, his hundreds into thousands, and his thousands into tens of thousands.

Here is another homely illustration which the country-bred nurse will be inclined to smile at! There is a hen's nest, and in it a solitary egg. If you stand a little distance away and watch it you will by and by see a timid hen, bent upon business, marching with cautious but steadfast steps in the direction of the nest. Wait but a little longer, and you will hear that same hen give utterance to a half-hysterical but wholly delighted cackling. You may permit her to go quietly away, and then, if you approach the nest you will find that your one egg has become two. Visit the spot again after five or six days, and there may be twenty or thirty eggs in the nest. It is not that the one hen has been so fruitful, but that other hens, hearing her cackling, have selected that particular nest for their own, and so the one nest egg has proved the nucleus from which a whole farmyard might now be stocked with cackling fowls.

The fifty thousand pounds of the Nurses' Pension Fund is looked upon by some as a final and completed achievement. It certainly is an achievement, just as the first hen's egg was an achievement, and one of which she was justly proud. But it is not to be considered in any sense a final achievement. On the contrary, it is the one seed corn which is to produce the golden ear; the one egg which is to attract many fowls to the nest, and finally to stock the whole farmyard with industrious and happy cacklers.

This article was commenced by a deliberately-expressed opinion about the value of money. What will money represent to aged and worn-out nurses? It will be to them comfort, health, life, and assured peace. It is not, therefore, for nurses to despise money. We shall agree with them in thinking that virtue and goodness are better than money; but we shall not agree with them in thinking that money is of little or no value. To nurses money is next in value to goodness and good sense. As Solomon says, it is "a defence"; a defence which it is practically impossible for nurses to be safe without.

The celebration at St. Thomas's Hospital on Tuesday gave definite shape to a thought which has been forming in the writer's mind for some few months. This is the thought: The fifty thousand pounds is a seed corn; it must grow to a golden ear. It is a nest egg; it must be the first of a nest-full, which shall finally stock the farmyard. But the seed grows to an ear within a limited time; and the one egg attracts a full nest within a limited time. The golden ear which is to spring from the single seed corn, and the full nest which is to succeed the single egg, is to be ten times fifty thousand pounds—five hundred thousand; half a million of money; and all this is to be accomplished, mainly by the nurses themselves, by the time that the young nurses of to-day have reached the qualifying age for their pensions. That is to say, if the founder of the Pension Fund lives, and the nurses will work one-tenth part as hard for themselves as he has worked, and is still willing to work, for them, the fifty thousand pounds will grow to half a million in twenty-five years. This is a practical thought which the nurses should take to heart. The writer asks them to take it to heart to-day.

